# Supplementary material for: TDP-43 represses cryptic exon inclusion in the FTD–ALS gene UNC13A
Source: Nature. 2022 Feb 23;603(7899):124–30. doi: 10.1038/s41586-022-04424-7 (PMC8891019; doi:10.1038/s41586-022-04424-7)
Supplement: Supplementary file 1 — This file contains Supplementary Tables 3–5 and Supplementary Notes 1–3. [file 41586_2022_4424_MOESM1_ESM.pdf]

nature portfolio

https://doi.org/10.1038/s41586-022-04424-7

Supplementary information

TDP-43 represses cryptic exon inclusion in the FTD–ALS gene *UNC13A*

In the format provided by the authors and unedited

nature portfolio

https://doi.org/10.1038/s41586-022-04424-7

Supplementary information

TDP-43 represses cryptic exon inclusion in the FTD–ALS gene *UNC13A*

In the format provided by the authors and unedited

SI Guide

TDP-43 represses cryptic exon inclusion in FTD/ALS gene *UNC13A*

X. Rosa Ma[1,†], Mercedes Prudencio[2,3,†], Yuka Koike[2,3,†], Sarat C. Vatsavayai[4,5], Garam Kim[1,6], Fred Harbinski[7], Adam Briner[1,15], Caitlin M. Rodriguez[1], Caiwei Guo[1], Tetsuya Akiyama[1], H. Broder Schmidt[8], Beryl B. Cummings[7], David W. Wyatt[7], Katherine Kurylo[7], Georgiana Miller[7], Shila Mekhoubad[7], Nathan Sallee[7], Gemechu Mekonnen[9,10], Laura Ganser[11], Jack D. Rubien[12], Karen Jansen-West[2], Casey N. Cook[2,3], Sarah Pickles[2,3], Björn Oskarsson[13], Neill R. Graff-Radford[13], Bradley F. Boeve[14], David S. Knopman[14], Ronald C. Petersen[14], Dennis W. Dickson[2,3], James Shorter[12], Sua Myong[9,10,11], Eric M. Green[7], William W. Seeley[4,5], Leonard Petrucelli[2,3,*], and Aaron D. Gitler[1,*]

Affiliations:

[1]Department of Genetics, Stanford University School of Medicine; Stanford, CA, USA

[2]Department of Neuroscience, Mayo Clinic; Jacksonville, FL USA

[3]Neuroscience Graduate Program, Mayo Clinic Graduate School of Biomedical Sciences; Jacksonville, FL, USA

[4]Department of Neurology, University of California San Francisco; San Francisco, CA, USA

[5]Department of Pathology, University of California San Francisco; San Francisco, CA, USA

[6]Neurosciences Interdepartmental Program, Stanford University School of Medicine; Stanford, CA, USA

[7]Maze Therapeutics; South San Francisco, CA USA

[8]Department of Biochemistry, Stanford University School of Medicine; Stanford, CA, USA

[9]Program in Cell, Molecular, Developmental Biology, and Biophysics, Johns Hopkins University, Baltimore, MD, USA

[10]Department of Biology, Johns Hopkins University, Baltimore, MD, USA

[11]Department of Biophysics, Johns Hopkins University, Baltimore, MD, USA

[12]Department of Biochemistry and Biophysics, Perelman School of Medicine, University of Pennsylvania, Philadelphia, PA, USA

[13]Department of Neurology, Mayo Clinic, Jacksonville, FL, USA

[14]Department of Neurology, Mayo Clinic; Rochester, MN, USA

[15]Clem Jones Centre for Ageing Dementia Research (CJCADR), Queensland Brain Institute (QBI), The University of Queensland, Brisbane, QLD, Australia

†These authors contributed equally to this work

*Corresponding authors. Email: agitler@stanford.edu or petrucelli.leonard@mayo.edu

Table of content:

Supplementary information:

This file contains Supplementary Tables 3-5, and Supplementary Notes 1-3.

Supplementary Figure 1: Uncropped images of immunoblots, PCR gels, and images of EMSA presented in this study.

Supplementary Table 1: List of genes alternatively spliced in the absence of TDP-43 identified by both MAJIQ and LeafCutter. The coordinates of the alternatively spliced regions are shown. *CADPS*, *SETD5* and *SYNE1* have multiple alternatively spliced regions. *APLP2*, *EPB41L1*, and *KIF3A* did not reach a ΔPSI larger than 10% according to LeafCutter, which can likely be attributed to the normalization in intron clusters. FDR-adjusted p-values are calculated as implemented in LeafCutter.

Supplementary Table 2: Detection of *STMN2* and *UNC13A* splice variants in bulk RNA-sequencing of patient tissues from the NYGC ALS Consortium cohort. a, Distribution of filtered samples and the percentage of samples retained from filtering in each category. We filtered for samples that had a TPM ⩾ 1.55, or at least 20 reads mapped to the "Exon 19-Exon 20" junction as samples suitable for *UNC13A* splice variant analysis (see Supplementary Note 3). **b, c,** Distribution of filtered samples containing the *STMN2* cryptic exon-containing splice variant **(b)** or the *UNC13A* cryptic exon-containing splice variant **(c).** And the percentage of filtered samples containing the *STMN2* splice variant **(b)** or *UNC13A* splice variant **(c)** in each category. The samples must have at least 2 reads spanning "Exon 1-Exon 2a" junction to be scored as containing the *STMN2* splice variant or at least 2 reads spanning either "Exon 20-CE" junction or "CE-Exon 21" to be scored as containing the *UNC13A* splice variant. **d,** Distribution of samples containing both the *STMN2* splice variant and the *UNC13A* splice variant, and the percentage of *UNC13A* splice variant-containing samples that alsocontain *STMN2* splice variant in each category.

Supplementary Table 6: List of all the primers used in the study

Supplementary Table 3: List of iPSC lines differentiated into motor neurons (iPSC-MNs)

ID	Cell line name	Sex	Age	Disease mutation	Source
iPSC-MN 1	GM25256	M	30	N/A	Coriell Institute
iPSC-MN 2	NDS00209	M	64	TDP43 G298S	NINDS
iPSC-MN 3	NDS00262	M	N/A	N/A	NINDS

Supplementary Table 4: List of sporadic FTLD-TDP and control cases used for the immunohistochemical analysis of TDP-43 and UNC13A cryptic exon inclusion

Case number	Age (years)	Sex	PMI (hrs)	Clinical diagnosis	Primary neuropathological diagnosis	ADNC
FTD-MND 1	72	M	6.7	bvFTD-MND	FTLD-TDP-B	not
FTD-MND 2	57	M	7.6	bvFTD-ALS	FTLD-TDP-B, MND	not
FTD-MND 3	66	M	12.1	bvFTD/nfvPPA, MND	FTLD-TDP-B, ALS	low
FTD-MND 4	65	F	8.5	bvFTD	FTLD-TDP-B; MND	low
Control 1	76	M	8.2	N/A	None	low
Control 2	67	F	19.4	N/A	None	not
Control 3	60	F	20.5	N/A	None	low

*Abbreviations: PMI – postmortem interval, bvFTD – behavioral variant frontotemporal dementia, ALS – Amyotrophic lateral sclerosis, MND – motor neuron disease, nfvPPA – non-fluent variant primary progressive aphasia, FTLD – frontotemporal lobar degeneration, ADNC - Alzheimer’s disease neuropathologic change.

Supplementary Table 5: List of RNA sequences used for MESA

RNA	Sequence
CE Ref.(G)	5'Cy3-aag**g**auggauggagagaugggugagu-*gccucgcugccgucgcca*-3'
CE Risk (C)	5'Cy3-aag**c**auggauggagagaugggugagu-*gccucgcugccgucgcca*-3'
Intron Ref.(U)	5'Cy3-aauggaugg**u**uggauaaauugaugggguggauggau-*gccucgcugccgucgcca*-3'
Intron Risk(G)	5'Cy3-aauggaugg**g**uggauaaauugaugggguggauggau-*gccucgcugccgucgcca*-3'
Repeat Ref ((GATG)$_4$)	5'Cy3-uggauggauggauggaugga-*gccucgcugccgucgcca*-3'
Repeat Risk ((GATG)$_9$)	5'Cy3-uggauggauggauggauggauggauggauggauggaug-*gccucgcugccgucgcca*-3'
Complementary 3' 18-mer	5'-biotin-*uggcgacggcagcgaggc*-3'Cy5

* Italicized sequences are part of the partial duplex and not the sequence of interest.
† Because *UNC13A* is mapped to the negative strand of genomic DNA, the reference and risk alleles used in this experiment are the reverse complement of the genotypes reported on dbSNP.

Supplementary Note 1: Using MAJIQ and LeafCutter to detect splicing variations

We used MAJIQ and LeafCutter to detect the splicing variations in the Liu et al.[1] RNA-Seq data because they are well-validated tools capable of detecting both *de novo* and canonical splicing variations. We did not intend to compare the two tools in our study; we were hoping to use the outputs from the tools to highlight genes of interest. We expected that the outputs from MAJIQ and Leafcutter would be different for the following reasons:

- Since detection of splicing variations using data from RNA sequencing is nontrivial, different data analysis tools tend to give different results[2].
- MAJIQ and LeafCutter use different modeling techniques. Even though both tools rely on junction spanning reads, the units of comparison used by the tools are different.
    - MAJIQ identifies and compares LSVs (local splicing variations), which are defined as splits (multiple edges) in a splice graph where several edges either come into or from a single exon reference exon[3].
    - LeafCutter detects intron inclusions and collapses all overlapping introns to an "intron cluster" for which the inclusion levels are normalized and quantified[4]. Namely, the intron cluster defined by Leafcutter is roughly equivalent to a cluster of neighboring LSVs. Additionally, after clustering the introns, instead of reporting the PSI values from each LSV, the intron inclusion levels in each LSV in the intron cluster are normalized and then quantified.
- Compared to MAJIQ, Leafcutter also does not detect alternative first and last exons, or intron retentions.

Because of the differences between the two tools and the goal of this study, we did not attempt to make the parameters used to run the two tools consistent. We would like to explain the rationale behind the following settings:

- We increased the value assigned to --min-intronic-cov at the build step of MAJIQ from the default 0.01 to 1. This parameter sets the minimum number of average reads per position in one of the bins in the introns to be considered to have sufficient coverage at that position. MAJIQ sets the size of the bin as the readlength of the RNA-Seq so that the size of bins are equivalent to the sizes of the junctions (because junctions are defined by reads that span the exon boundaries). Since the RNA-Seq libraries from Liu et al.[1] were prepared from nuclei, it is likely that they may include more reads mapped to the introns than what would normally be expected. However, setting --min-intronic-cov to 1 is an arbitrary decision. None of the genes presented in Fig. 1b has intron retention because such variations could not be detected by LeafCutter.
- At the time of running the software (commit 249fc26 on https://github.com/davidaknowles/leafcutter), a script (filter_cs.py) provided by LeafCutter is used in "Step1 Converting bams to juncs". The script filters for reads that span introns longer than 50 bp and have at least 6 nt overhangs that are mapped into each

spanning exon. MAJIQ does not explicitly filter for reads based on the length of the intron they span and the length of the overhangs. Moreover, during the alignment stage using STAR, this problem is handled by requiring reads that span unannotated junctions to have a minimum overhang of 8nt (--alignSJoverhangMin 8; this is consistent with the ENCODE standard options).

Because the tools use different units for transcript variations (LSVs vs. intron clusters), we also do not think the ΔPSIs from the two tools are directly comparable. While MAJIQ focuses on the PSIs at an LSV, the PSIs in LeafCutter are normalized to the entire intron cluster. We also cannot compare the p-values from the two tools because MAJIQ does not produce a p-value but a Bayesian posterior for P(ΔPSI> C). While ΔPSI gives us the information about the magnitude of changes, p-value, or in the case of MAJIQ, P(ΔPSI > C), shows the tool's belief in the splicing changes. In our case, we decided that the confidence in changes is more interesting than the magnitude of changes, since small changes in transcriptome could be sufficient to lead to functional changes. Specifically, when filtering for LeafCutter outputs with an adjusted p-value smaller than 0.05, we get 139 unique genes. To get a similar number of genes from the output of MAJIQ, we set the threshold C as 0.1, which is more permissive than the default setting 0.2. The filtered MAJIQ outputs contain 198 unique genes. The outputs from the two tools are event matched and the coordinates of the corresponding regions are provided in Supplementary Table 1. There are 66 overlapping genes, among which CADPS contains 3 regions, SETD5 contains 2 regions, and SYNE1 contains 2 regions that go through differential splicing. Only 3 genes (APLP2, EPB41L1 and KIF3A) from the outputs of LeafCutter did not reach a ΔPSI larger than 10%. However, this can likely be attributed to the normalization in intron clusters.

While looking at the overlapped list boosts our confidence in the outputs, we still think it is valuable to look at genes that did not make it to the list (Fig. 1b) using the interactive interface provided by each of them. For example, only the output from MAJIQ contains genes that have alternative first and final exons or intron retentions. However, it is challenging to present the outputs from the two pipelines in a spreadsheet due to the complexity of splicing events, and such format may not be very informative. Therefore, we encourage readers to explore the outputs using the interactive interface provided by each of the pipelines. Instructions to visualize the results are provided in the GitHub repository (see Code Availability). Furthermore, the splice variation should also be verified using visualization tools such as IGV and experimental methods such as qRT-PCR, if possible. Considering that the average length of a human exon is ~300 bp[5], with the most commonly used RNA-Seq read length being 150 bp, most of the junction spanning reads do not span more than one exon. In other words, the splice variations that we can detect are all local. While there are isoform-based tools such as Diffsplice and Cuffdiff2[2], the best way to detect isoform variants, especially de novo isoform variants, would be to use long-read sequencing technology. That said, existing tools are still great resources for analyzing existing RNA-seq data.

(This supplementary note is written with the help of the comparison done by Dr. Yoseph Barash, posted on https://biociphers.wordpress.com/2017/12/11/leafcutter-vs-majiq-and-comparing-differential-splicing-algorithms/)

Supplementary Note 2: Using MAJIQ and LeafCutter to detect splicing variations

From the splicing analysis using RNA-Seq data from Liu et al (Fig. 1a), we found that depletion of TDP-43 introduces two alternative 3' splice acceptor sites in intron 20-21: one near chr19:17642591($\Delta\Psi$=0.05246) and the other at chr19:17642541($\Delta\Psi$=0.49267). They share the same alternative 5' splice donor site near chr19:17642414 ($\Delta\Psi$=0.7763) (Extended Data Fig. 1a). This creates two different cryptic exons (Extended Data Fig. 1e). Since we saw much higher usage of the chr19:17642541 3' splicing acceptor, we focused on the 128 bp cryptic exon (CE) defined by this 3' splice acceptor and the alternative 5' splice acceptor. The other version of the cryptic exon (hg38; chr19: 17642414-17642591) is 50 bp longer than the more abundant version (CE-2) (Extended Data Fig. 1e). RT-PCR amplifying the exon 20-exon 21 region (Extended Data Fig. 3a) indicates that there is also a version of the cryptic exon which uses the same alternative 5' splice donor but includes the entire intron between exon 20 and CE (CE-3) (hg38; chr19: 17642844-17642414). Due to their similar sizes, CE and CE-2 resulted in a single band on the gel. It is likely that CE-3 was not observed in the splicing analysis (Fig. 1a) because of the stringent parameter choice. All three cryptic exons were detected in the mini-gene experiment (Fig. 4f). CE-2 is 178 bp and CE-3 is 431bp, indicating that, similar to CE, both CE-2 and CE-3 would introduce a pre-mature stop codon and lead to dysregulation of UNC13A protein expression. The primer pair we used for RT-qPCR for this study spans the CE-exon 21 junction, which is the same as CE-2-exon 21 and CE-3-exon 21 junctions. Therefore, the primer pair can detect all three versions of the cryptic exon.

Supplementary Note 3: Detection of cryptic splicing event in bulk RNA sequencing

It is estimated that the non-neuron to neuron ratio (nNNR) of the human spinal cord, the cerebral cortex (gray and white matter combined), and the cerebellum are 6.5, 4.32 and 0.29, respectively[6]. Because of the heterogeneity of the tissues, relatively low expression level of UNC13A (average TPM of STMN2 across all the samples is 49.6 vs. 10.5 of UNC13A), and the low abundance of cells affected by TDP-43 pathology (7.05% of all neuronal nuclei and <2% of all cells in the neocortex according to Liu et al. [1]), it is possible that not all sampled tissues have enough UNC13A transcripts for us to detect the splice variants. Additionally, the coding sequence of UNC13A (ENST00000551649.5) is 6052 bp, which is almost twice as long as the length of an average coding sequence[5], and the RNA-Seq coverages of mRNA transcripts are often not uniformly distributed[7]. In light of these challenges, we used the following criteria to filter for tissue samples to include in the analysis:

1. To filter for tissue samples with sufficient relative abundance of UNC13A transcripts for detecting the (128 bp) CE-containing variants, the UNC13A TPM needs to be at least 1.55. We observed that samples with at least 2 reads spanning either "Exon 20-CE" junction or "CE-Exon 21" junction have at least UNC13A TPM = 1.55. This indicates that if we want to detect reads that span the cryptic exon junctions, there should be at least 1.55 UNC13A transcripts per million of sequenced full length transcripts. A TPM lower than that 1.55 UNC13A may indicate that the tissue samples contain too few neuronal cells for us to detect the splice variants, which would only be in neurons that are affected by TDP-43 pathology.

2. To ensure that there is sufficient coverage at "Exon 20-Exon 21", "Exon 20-CE", or "CE-Exon 21" junctions, there need to be at least 20 reads mapped to the "Exon 19-Exon 20". Instead of directly looking at reads mapped to the junctions of interest, we used the number of reads spanning the closest junction upstream of "Exon 19- Exon 20" as a proxy. The "Exon 19-Exon 20" junction is included in both the canonical isoform and the splice variant, and there is a strong correlation (Pearson's r = 0.99) between the numbers of reads mapped to "Exon 19- Exon 20" junction and "Exon 20-Exon 21" junction. We observed that samples that have at least 2 reads spanning either "Exon 20-CE" junction or "CE-Exon 21" junction have at least 20 reads spanning the "Exon 19- Exon 20" junction. This indicates that the coverage at the "Exon 19-Exon 20" junction may need to be at least 20 to have sufficient coverage at the junctions of interest for us to detect the splice variants.

3. Using the parameters above, we retained 1151 of the original 1658 samples. Most of the tissue samples filtered out are from spinal cords (Supplementary Table 2a), possibly due to the high nNNR ratio and the lower expression level of UNC13A in the spinal cord compared to other brain regions.

We also detected reads that span the exon 1 and the cryptic exon of STMN2 using the 1151 samples. The results are included in Supplementary Table 2b.

References:

1. Liu, E. Y. *et al.* Loss of Nuclear TDP-43 Is Associated with Decondensation of LINE Retrotransposons. *Cell Rep.* (2019) doi:10.1016/j.celrep.2019.04.003.
2. Mehmood, A. *et al.* Systematic evaluation of differential splicing tools for RNA-seq studies. *Brief. Bioinform.* **21**, 2052–2065 (2020).
3. Vaquero-Garcia, J. *et al.* A new view of transcriptome complexity and regulation through the lens of local splicing variations. *Elife* (2016) doi:10.7554/eLife.11752.
4. Li, Y. I. *et al.* Annotation-free quantification of RNA splicing using LeafCutter. *Nat. Genet.* (2018) doi:10.1038/s41588-017-0004-9.
5. Piovesan, A., Caracausi, M., Antonaros, F., Pelleri, M. C. & Vitale, L. GeneBase 1.1: a tool to summarize data from NCBI gene datasets and its application to an update of human gene statistics. *Database (Oxford).* **2016**, baw153 (2016).
6. Bahney, J. & von Bartheld, C. S. The Cellular Composition and Glia-Neuron Ratio in the Spinal Cord of a Human and a Nonhuman Primate: Comparison With Other Species and Brain Regions. *Anat. Rec. (Hoboken).* **301**, 697–710 (2018).
7. Hansen, K. D., Brenner, S. E. & Dudoit, S. Biases in Illumina transcriptome sequencing caused by random hexamer priming. *Nucleic Acids Res.* (2010) doi:10.1093/nar/gkq224.
