# Supplementary material for: TDP-43 represses cryptic exon inclusion in the FTD–ALS gene UNC13A
Source: Nature. 2022 Feb 23;603(7899):124–30. doi: 10.1038/s41586-022-04424-7 (PMC8891019; doi:10.1038/s41586-022-04424-7)
Supplement: Supplementary file 2 — Reporting Summary [file 41586_2022_4424_MOESM2_ESM.pdf]

nature portfolio

Corresponding author(s): Aaron D Gitler, Leonard Petrucelli

Last updated by author(s): Dec 11, 2021

Reporting Summary

Nature Portfolio wishes to improve the reproducibility of the work that we publish. This form provides structure for consistency and transparency in reporting. For further information on Nature Portfolio policies, see our Editorial Policies and the Editorial Policy Checklist.

Statistics

For all statistical analyses, confirm that the following items are present in the figure legend, table legend, main text, or Methods section.

n/a	Confirmed	
☐	☒	The exact sample size (*n*) for each experimental group/condition, given as a discrete number and unit of measurement
☐	☒	A statement on whether measurements were taken from distinct samples or whether the same sample was measured repeatedly
☐	☒	The statistical test(s) used AND whether they are one- or two-sided<br>*Only common tests should be described solely by name; describe more complex techniques in the Methods section.*
☐	☒	A description of all covariates tested
☐	☒	A description of any assumptions or corrections, such as tests of normality and adjustment for multiple comparisons
☐	☒	A full description of the statistical parameters including central tendency (e.g. means) or other basic estimates (e.g. regression coefficient) AND variation (e.g. standard deviation) or associated estimates of uncertainty (e.g. confidence intervals)
☐	☒	For null hypothesis testing, the test statistic (e.g. *F*, *t*, *r*) with confidence intervals, effect sizes, degrees of freedom and *P* value noted<br>*Give P values as exact values whenever suitable.*
☒	☐	For Bayesian analysis, information on the choice of priors and Markov chain Monte Carlo settings
☒	☐	For hierarchical and complex designs, identification of the appropriate level for tests and full reporting of outcomes
☐	☒	Estimates of effect sizes (e.g. Cohen's *d*, Pearson's *r*), indicating how they were calculated

Our web collection on statistics for biologists contains articles on many of the points above.

Software and code

Policy information about availability of computer code

Data collection	N/A
Data analysis	trimmomatic (0.39), FastQC (v0.11.9), STAR v2.7.3a, RSEM v1.3.1, R v4.0.0 and R v4.0.2, DESeq2(v1.28.1), MISO(misopy 0.5.4),, VCFtools (0.1.16), BCFtools (1.8), Prism 8; https://github.com/rosaxma/TDP-43-UNC13A-2021

For manuscripts utilizing custom algorithms or software that are central to the research but not yet described in published literature, software must be made available to editors and reviewers. We strongly encourage code deposition in a community repository (e.g. GitHub). See the Nature Portfolio guidelines for submitting code & software for further information.

Data

Policy information about availability of data

All manuscripts must include a data availability statement. This statement should provide the following information, where applicable:

- Accession codes, unique identifiers, or web links for publicly available datasets
- A description of any restrictions on data availability
- For clinical datasets or third party data, please ensure that the statement adheres to our policy

The amplicon sequencing data has been deposited in the Gene Expression Omnibus (GEO) at GSE182976. RNA-Seq data for splicing analysis is available at GSE126543. RNA-Seq data generated by NYGC ALS Consortium cohort is available at GSE137810, GSE124439, GSE116622, and GSE153960

Field-specific reporting

Please select the one below that is the best fit for your research. If you are not sure, read the appropriate sections before making your selection.

☒ Life sciences ☐ Behavioural & social sciences ☐ Ecological, evolutionary & environmental sciences

For a reference copy of the document with all sections, see nature.com/documents/nr-reporting-summary-flat.pdf

Life sciences study design

All studies must disclose on these points even when the disclosure is negative.

Sample size	No sample size calculation was performed; for in vitro experiments, sample sizes were choses such that statistical significance could be confidently established; for analysis using patient data, sample sizes were chosen based on data availability.
Data exclusions	No data from in vitro experiments were excluded. The data exclusion criteria for the following experiments/analyses were pre-established. For the in situ hybridization experiment using postmortem brain samples, subject were excluded if they had a known disease-causing mutation, post-mortem interval ≥ 24 h, Alzheimer's disease neuropathologic change > low, Thal amyloid phase > 2, Braak neurofibrillary tangle stage > 4, CERAD neuritic plaque density > sparse, and Lewy body disease > brainstem predominant. For the multiple linear regression analysis, we excluded one subject who does not carry the haplotypes we were studying. In the analysis that quantifies UNC13A splice variants in bulk RNA, we exclude samples whose UNC13A expression was too low for us to detect the splice variants.
Replication	All the experiments presented in the studies have been successfully replicated in independent cell cultures. The results of the experiments contributing to the main findings have been replicated in separate studies.
Randomization	Randomization is not applicable to cell culture experiments. Randomization is also not applied to the experiments that involve human postmortem tissues.
Blinding	Investigators were not blinded to the identity of the samples but findings have been replicated by independent investigators in multiple laboratories.

Reporting for specific materials, systems and methods

We require information from authors about some types of materials, experimental systems and methods used in many studies. Here, indicate whether each material, system or method listed is relevant to your study. If you are not sure if a list item applies to your research, read the appropriate section before selecting a response.

Materials & experimental systems

n/a	Involved in the study	
☐	☒	Antibodies
☐	☒	Eukaryotic cell lines
☒	☐	Palaeontology and archaeology
☒	☐	Animals and other organisms
☐	☒	Human research participants
☒	☐	Clinical data
☒	☐	Dual use research of concern

Methods

n/a	Involved in the study	
☒	☐	ChIP-seq
☒	☐	Flow cytometry
☒	☐	MRI-based neuroimaging

Antibodies

Antibodies used	HRP-conjugated anti-mouse IgG (H+L) (Fisher 62-6520), HRP-conjugated anti-rabbit IgG (H+L) (Life Technologies 31462)
Validation	HRP-conjugated anti-mouse IgG (H+L) (https://www.thermofisher.com/antibody/product/Goat-anti-Mouse-IgG-H-L-Secondary-Antibody-Polyclonal/62-6520). Extracts from multiple cell lines were first probed with appropriate mouse monoclonal antibodies and then probed with HRP-conjugated anti-mouse IgG (H+L) to verify specificity.<br>HRP-conjugated anti-rabbit IgG (H+L) (Life Technologies 31462) (https://www.thermofisher.com/antibody/product/Goat-anti-Rabbit-IgG-H-L-Cross-Adsorbed-Secondary-Antibody-Polyclonal/31462). Extracts from multiple cell lines were first probed with appropriate rabbit polyclonal antibodies and then probed with HRP-conjugated anti-rabbit IgG (H+L) to verify specificity.

Eukaryotic cell lines

Policy information about cell lines

Cell line source(s)	SH-SY5Y, HEK293T: ATCC; iPSCs: Coriell Repository

Authentication	Cell line authentication was performed by the supplier.<br>SH-SY5Y: STR profiling.<br>iPSC lines: GM25256 (karyotypic analysis), NDS00262(96 SNP assay), NDS00209(96 SNP assay).<br>HEK293T: STR profiling.
Mycoplasma contamination	Cells were not test for mycoplasma contamination by our labs.
Commonly misidentified lines<br>(See ICLAC register)	Cell lines were not listed in ICLAC register.

Human research participants

Policy information about studies involving human research participants

Population characteristics	Mayo Clinic Brain Bank: patients with FTLD-TDP and cognitively normal control individuals; UCSF: see Supplementary Table 4
Recruitment	Written informed consent was obtained before study entry from all subjects or their legal next of kin if they were unable to give written consent, and biological samples were obtained with Mayo Clinic Institutional Review Board (IRB) approval.
Ethics oversight	Mayo Clinic Institution Review Board and Ethics Committee, UCSF Committee on Human Research

Note that full information on the approval of the study protocol must also be provided in the manuscript.
