# Supplementary figures and images for: TDP-43 represses cryptic exon inclusion in the FTD–ALS gene UNC13A
Source: Nature. 2022 Feb 23;603(7899):124–30. doi: 10.1038/s41586-022-04424-7 (PMC8891019; doi:10.1038/s41586-022-04424-7)

**a**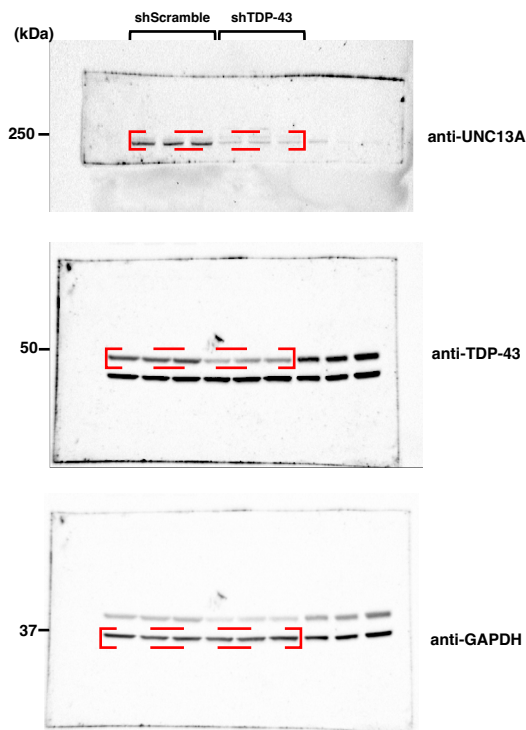**b**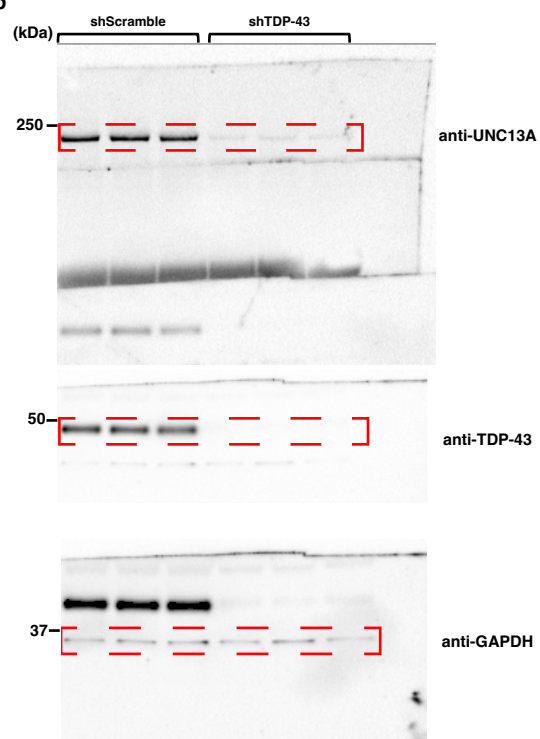**c**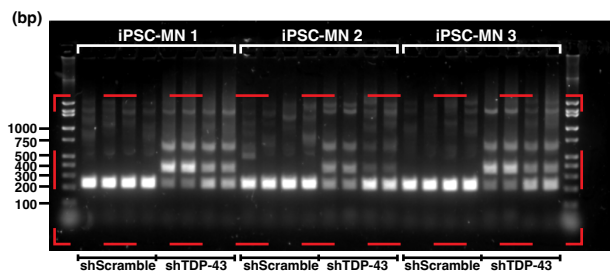**d**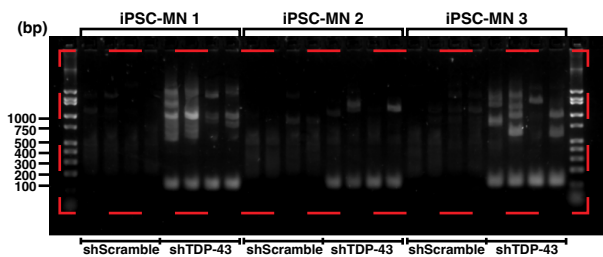**e**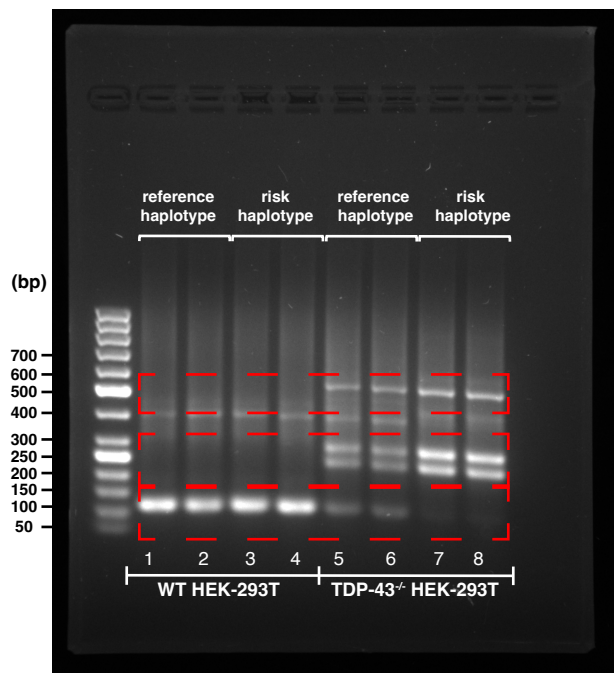**f**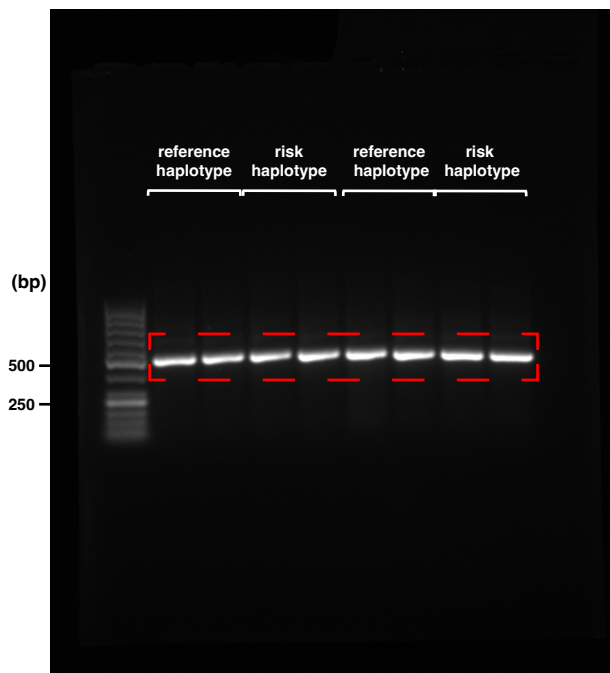

g

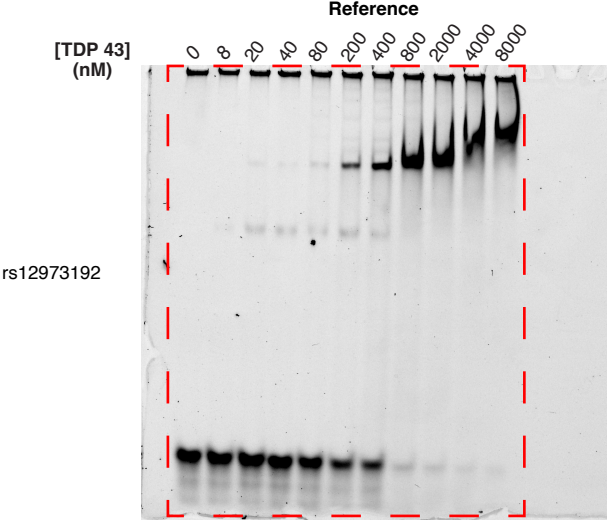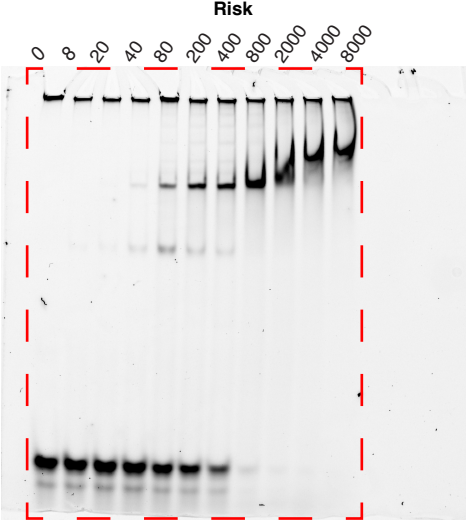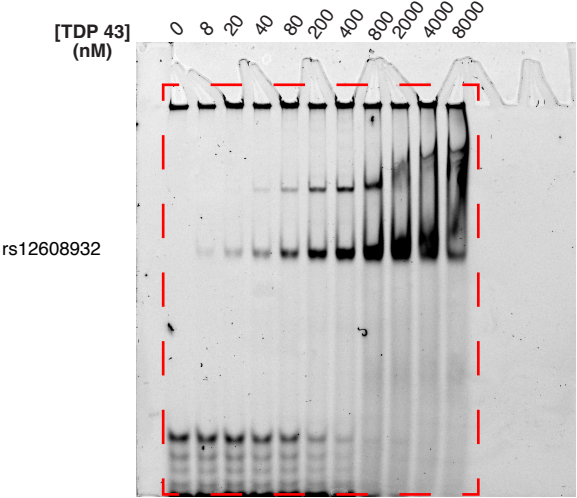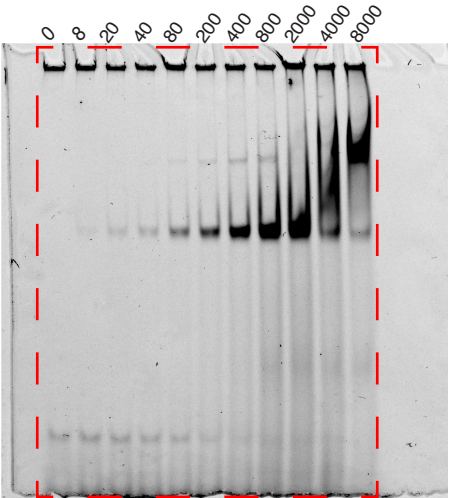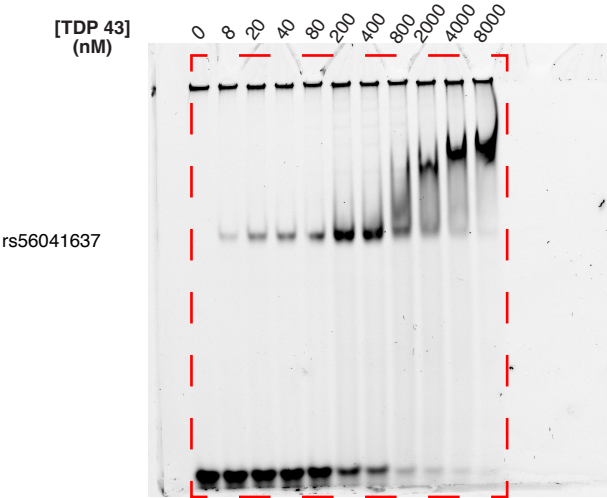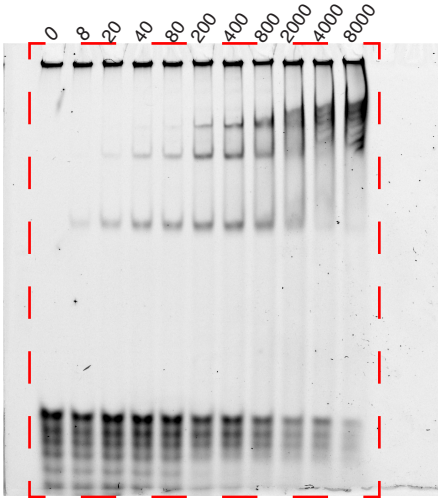

Supplement: Supplementary file 3 — Uncropped images of immunoblots, PCR gels, and images of EMSA presented in this study. [file 41586_2022_4424_MOESM3_ESM.pdf]
